# Food Iminosugars and Related Synthetic Derivatives Shift Energy Metabolism and Induce Structural Changes in Colon Cancer Cell Lines

**DOI:** 10.3390/foods14101713

**Published:** 2025-05-12

**Authors:** Thomas Montebugnoli, Charlotte Grootaert, Alessandra Bordoni, Andreja Rajković, Elien Alderweireldt, Jeltien Rombaut, Sofie L. De Maeseneire, John Van Camp, Maarten Lieven De Mol

**Affiliations:** 1 Department of Agricultural and Food Sciences (DISTAL), Alma Mater Studiorum—University of Bologna, Piazza Goidanich, 60, 47521 Cesena, Italy; thomas.montebugnoli2@unibo.it (T.M.); alessandra.bordoni@unibo.it (A.B.); 2 Department of Food Technology, Safety and Health, Faculty of Bioscience Engineering, Ghent University, Coupure Links 653, B-9000 Ghent, Belgium; charlotte.grootaert@ugent.be (C.G.); andreja.rajkovic@ugent.be (A.R.); elien.alderweireldt@ugent.be (E.A.); 3 Interdepartmental Centre of Agrifood Industry Research, Alma Mater Studiorum—University of Bologna, Via Quinto Bucci, 336, 47521 Cesena, Italy; 4 Department of Biotechnology, Faculty of Bioscience Engineering, Ghent University, Coupure Links 653, B-9000 Ghent, Belgium; jeltien.rombaut@ugent.be (J.R.); sofie.demaeseneire@ugent.be (S.L.D.M.); maarten.demol@ugent.be (M.L.D.M.)

**Keywords:** iminosugars, colon cancer, 3D spheroid culture, Caco-2, HCT-116, mitochondrial respiration, glycolysis, glycosidase inhibition

## Abstract

Iminosugars have a carbohydrate-like backbone in which the ring oxygen is replaced by nitrogen. They are naturally found in foods such as rice, buckwheat, mulberries, and fermented vegetables, and are reported to exert anti-hyperlipidemic and anti-hyperglycemic effects due to the inhibition of cellular glycosidases. This mechanism suggests their potential role in cancer treatment and prevention. In this study, two natural iminosugars, D-fagomine (FGM) and 1-deoxynojirimycin (DNJ), and their synthetic derivatives were screened for potential anticancer properties using Caco-2 and HCT-116 cells as models for the early and late stages of colon cancer, respectively. Iminosugars were found to decrease cell viability, with effects varying based on the type of iminosugar, cell type, growth condition (glucose concentration), exposure time (1 vs. 13 days), and tissue architecture (monolayer vs. spheroid). The combined use of innovative techniques, such as IncuCyte^®^ live cell imaging and Seahorse real-time cellular metabolic analysis, and microscopic observation after staining enabled us to detect changes in substrate utilization for energy metabolism, including increased glycolysis and alterations in lipid and glycogen stores. The evidence that iminosugars, both natural and synthetic, influence cellular bioenergetics paves the way for their potential use in various applications, including cancer treatment.

## 1. Introduction

Iminosugars, also known as azasugars, are small organic molecules that mimic carbohydrates. These compounds can be found in nature or synthesized in monocyclic or bicyclic forms. The distinguishing feature of iminosugars compared to common sugars is the presence of nitrogen instead of oxygen in the ring structure. In nature, there are five main classes of iminosugars: indolizidines, piperidines, nortropanes, pyrrolizidines, and pyrrolidines. Synthetic iminosugars, on the other hand, encompass a wide variety of ring structures [1].

Iminosugars act as potent inhibitors of glycosidases, enzymes responsible for breaking glycosidic bonds in oligosaccharides and glycoconjugates. Additionally, they effectively inhibit glycosyltransferases, enzymes that catalyze the formation of glycosidic bonds from sugar donors with a leaving group in the anomeric position [2]. Iminosugars are found in various plants, aiding in their defense against destruction by animals or pathogens, and thus often end up in foods. Once ingested, iminosugars can perform several beneficial actions in the body. For example, the stevia plant contains iminosugars like steviamine, which inhibit glycosidases and show promising antimetastatic activity. Some cucumber varieties possess idoBR1, an iminosugar with potent anti-inflammatory activity. Another example is the Indian Ayurvedic medicinal plant Gymnema sylvestre, which may restore insulin responses and beta-cell health over time. Despite these examples, iminosugars have often been overlooked, and many are believed to be yet undiscovered in food plants. For instance, potatoes contain nortropan calistegins, potent inhibitors of glucosidase and galactosidase. Rice and buckwheat contain D-fagomine (FGM), which can inhibit a wide range of enzymes, although the presence of iminosugars in rice may depend on the variety [3]. Iminosugars from rice and potato are stable after cooking and are excreted in urine once ingested.

Clinical studies have shown that FGM can control postprandial glycemic response and improve immune responses in the presence of opportunistic microorganisms [4]. FGM was first isolated as a natural product in 1974 from Japanese buckwheat (*Fagopyrum esculentum Moench*) seeds, leading to the discovery of its inhibitory activities against *α*-glucosidase and *β*-galactosidase in mammals. As studies on FGM intensified, a regioisomeric analogue, Isofagomine (IFGM), was synthesized in 1994 and proved to be a potent glucosidase inhibitor. IFGM has been used in the treatment of Gaucher disease as an active-site-specific pharmacological chaperone (ASSPC) strategy [5].

One of the most well-known naturally occurring iminosugars is 1-deoxynojirimycin (DNJ), an analog of D-glucose with a nitrogen atom substituted for oxygen in the ring. DNJ is found in various parts of mulberry trees and is known for its antidiabetic, antiviral, and antitumor activities. It is the basis of the diabetes drug Glyset^TM^, also known as Miglitol (MGTL), due to its function as a potent inhibitor of various glucosidases. In the intestine, DNJ and MGLT inhibit digestive glucosidases such as maltase, isomaltase, and sucrase. Thus, compounds like DNJ can be used therapeutically in the oral treatment of non-insulin-dependent diabetes mellitus (type II) due to their inhibitory ability against β-glucosidases [3]. Besides mulberries, DNJ is produced by several *Bacillus* and *Streptomyces* strains, such as *Bacillus subtilis* MORI and *B. amyloliquefaciens*, isolated from traditional Korean fermented foods like *Chungkookjang. B. amyloliquefaciens* has also been isolated from traditional Korean fermented cabbage, Kimchi, and several other fermented foods. *B. atrophaeus* has been identified in fermented tofu, *B. subtilis* in the traditional Chinese fermented Okara, Meitauza, and *B. velezensis* in traditional Korean fermented soybean paste, Doenjang. DNJ inhibits glucosidases I and II, which are involved in N-linked glycosylation of secretory proteins [6,7]. N-(n-Nonyl)deoxynojirimycin (NN-DNJ) is a derivative of DNJ with a nonyl group attached to the nitrogen atom, increasing its hydrophobicity. This modification enhances its interaction with biological membranes, altering membrane permeability and structural integrity [8]. Computational analysis by Thirumal Kumar et al. (2019) suggested that NN-DNJ could act as a pharmacological chaperone to enhance β-glucocerebrosidase activity in patients with specific mutations, paving the way for potential applications in the treatment of Gaucher’s disease [9]. Another DNJ derivative is 2,3,4,6-Tetra-O-benzyl-(+)-1-deoxynojirimycin (TBN-DNJ), widely employed as a protected intermediate for the synthesis of N-substituted DNJ derivatives, enabling selective functionalization at the nitrogen position [10].

So far, iminosugars have mainly been studied as therapeutics to improve metabolic health by acting on intestinal enzymes. However, another application in which glycosidases play an important role is cancer. Glycosidase activity is increased in tumor cells and may aid in tumor cell shedding, local invasion, and structural changes in cell surface glucoconjugates. These changes increase attachment to endothelial cells and metastasis [11]. Besides their ability to modulate protein glycosylation, iminosugars may inhibit specific glycosidases involved in cell proliferation and interfere with the altered metabolic processes of cancer cells. While further research is needed to optimize their selectivity and reduce potential side effects, their role in regulating tumor-related biochemical pathways represents a growing area of interest in oncological research [12]. Yet, the effect of iminosugars on these pathways remains relatively unexplored.

**Figure 1:**
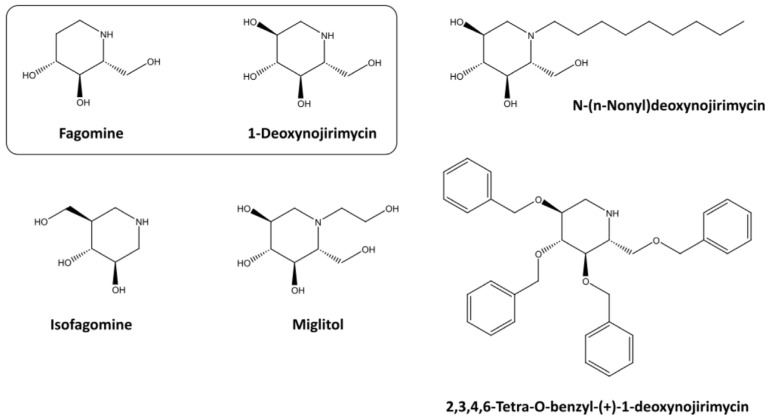
Differences in chemical structure between natural iminosugars (boxed) and their synthetic derivatives. Isofagomine is derived from Fagomine, while 1-Deoxynojirimycin serves as the core for the synthetic derivatives Miglitol (Glyset^®^), N-(nonyl)deoxynojirimycin, and 2,3,4,6-Tetra-O-benzyl-(+)-1-deoxynojirimycin.

In this study, we aim to compare the potential of different food-related and synthetic iminosugars on intestinal cell processes that may be related to their anticancer effects (Figure 1). We will focus on naturally derived iminosugars, such as FGM and DNJ, and their synthetic derivatives, IFGM, MGTL, NN-DNJ, and TBN-DNJ. As a case study, we investigated colon cancer, as the intestine is one of the first exposure sites of food-related compounds. Moreover, in vivo studies have shown that intestinal glycosidase activity is implicated in tumor progression in the colon, suggesting that their inhibition may represent a promising therapeutic strategy. This makes intestinal tissue a relevant model for exploring both the local enzymatic effects and potential anticancer mechanisms of iminosugars. In vitro experiments were performed under different nutritional conditions (glucose-rich and glucose-depleted medium), in two different cell lines representing different stages of colon cancer (Caco-2 and HCT-116 cells as early and later stages, respectively), and in both 2D monolayer and 3D spheroid morphology, the latter being a better reproduction of the tumor architecture and nutrient gradient [13]. Cell viability was assessed using standard microscopic and biochemical tools, bioenergetic parameters such as mitochondrial health and glycolytic capacity were further explored using the Seahorse Extracellular Flux (XF) Analyzer, and the effect on tissue organization was investigated by the staining of intracellular nutrient stores.

## 2. Materials and Methods

### 2.1. Chemicals

Iminosugars were purchased from the company Biosynth (Bratislava, Slovakia). Dulbecco’s modified Eagle’s medium (DMEM), DMEM without glucose, penicillin, streptomycin, and Dulbecco’s phosphate-buffered saline (DPBS) were purchased from Gibco (Fisher Scientific, Merelbeke, Belgium). Trypan blue and Fetal Bovine Serum (FBS) were obtained from Thermo Fisher Scientific, whereas XF base medium was purchased from Agilent (Ghent, Belgium). Seahorse plates, Seahorse cartridges, Seahorse calibrant, and Mito Stress assay kits were all obtained from Agilent. Cell culture plates with 96 wells and spheroid plates were purchased from Greiner (Vilvoorde, Belgium) and S-BIO (Neuss, Germany).

### 2.2. Cell Culture and Exposure

Caco-2 and HCT-116 cells (ATCC, Molsheim, France) were cultured in DMEM with 4.5 g·L^−1^ of glucose, supplemented with 10% heat-inactivated and sterile-filtered FBS, 1% non-essential amino acids, and 1% penicillin/streptomycin, at 37 °C and 10% CO_2_. The medium was refreshed every two or three days. Upon 80% confluency, cells were trypsinized, counted using trypan blue and a Bürker counting chamber, and seeded in plates. For the monolayer experiments, cells were seeded in 96-well plates at 2 × 10^4^ cells per well and cultured in two different media: glucose-rich DMEM (4.5 g·L^−1^ glucose and 10% FBS) or glucose-depleted DMEM (no glucose and 2% FBS). The HCT-116 cell line was used further in spheroid experiments (3D culture), where cells were seeded at 5 × 10^3^ cells per well in a 384-well plate. Cells were treated with different iminosugar concentrations (25–50 μM) for 1 or 13 days.

### 2.3. Resazurin Assay

The activity of cellular dehydrogenases as a measure for mitochondrial respiration was quantified with the resazurin assay, where metabolically active cells reduce resazurin, a non-fluorescent blue dye, into resorufin, which is fluorescent and pink in color. The resazurin stock solution (1 mg·mL^−1^ in distilled water) was diluted 1:100 *v*/*v* in DMEM (without FBS), resulting in a final concentration of 10 μg·mL^−1^. The plate was incubated at 37 °C for 2 h, after which fluorescence (λexc/λem = 560/590 nm) was measured in a 96-well black plate.

### 2.4. Sulforhodamine B Assay

The sulforhodamine B (SRB) assay was used as an indirect indicator of cell proliferation. The amount of SRB bound to the cells, measured spectrophotometrically, is proportional to the number of cells present in the sample. After the resazurin assay, cells were fixed by adding 50% TCA solution (1:4 *v*/*v*) to the medium and incubated at 4 °C for at least one hour. Next, cells were washed 5 times with tap water, and 50 µL of SRB solution was added to each well. After 30 min of incubation, the plates were washed 5 times with 1% acetic acid solution. The protein-bound SRB staining was solubilized by adding 200 µL of a 10 mM Tris solution, and the absorbance was measured at 490 nm.

### 2.5. IncuCyte Live Cell Imaging

IncuCyte^®^ is an advanced live-cell imaging system for acquiring and analyzing images directly inside an incubator, thereby providing real-time information on cell growth, metabolism, and other cellular dynamics to ensure optimal conditions for experiments even over extended times. Plates containing iminosugar-treated cells were placed in a 37 °C incubator, and cell growth was monitored over time. For Caco-2 and HCT-116 cells seeded in 96-well plates (2D culture), 4 images per well were acquired every 4 h. In the case of the spheroid experiment in 384-well plates (3D culture), one image per well was acquired every 12 h.

### 2.6. Seahorse Extracellular Flux Assays

The Agilent Seahorse XFe96 Extracellular Flux Analyzer was used in combination with the Agilent Seahorse XF Cell Mito Stress Test kit to assess mitochondrial function by targeting specific points in the electron transport chain (ETC). Oligomycin (OM), a complex V inhibitor, reduces electron flow through the ETC by blocking ATP synthase, leading to a decreased oxygen consumption rate and a corresponding decline in mitochondrial respiration, and hence mitochondrial ATP production. Carbonyl cyanide-p-trifluoromethoxyphenylhydrazone (FCCP), a protonophore, disrupts the mitochondrial membrane potential by collapsing the proton gradient, thereby allowing unrestricted electron flow through the ETC, resulting in maximal oxygen consumption by complex IV. The oxygen consumption rate stimulated by FCCP is used to calculate spare respiratory capacity, the difference between maximal and basal respiration, providing insights into mitochondrial efficiency and ATP synthesis capacity [14]. By measuring the oxygen consumption rate and extracellular acidification rate, basal respiration, maximal respiration, ATP production, proton leakage, non-mitochondrial respiration, and respiratory spare capacity were calculated according to the manufacturers’ protocols. Practically, the day before the experiment, cartridges were hydrated in calibration solution, placed in a sealed plastic bag containing moist paper to prevent dehydration, and incubated at 37 °C for 24 h. On the following day, the 96-well Agilent Seahorse spheroid plates were prepared by adding 30 µL of poly-D-lysine to each well and incubated for 20 min. Each well was then washed twice with 200 µL of water. Next, the wells were rinsed with 200 µL of XF DMEM Seahorse supplemented with pyruvate, glucose, and glutamine in a 100:1:1:1 ratio and subsequently filled with 175 µL of the same medium pre-warmed to 37 °C. Finally, spheroids were individually collected using a pipette and transferred from the 384-well plate to the 96-well Agilent Seahorse plates. Subsequently, 22 µL of OM (1 µM final concentration) was added to injection port A of the cartridge, 22 µL of FCCP (0.5 µM final concentration) to port B, and 25 µL of Rotenone/Antimycin A (R/AM, 0.5 µM final concentration) to port C. The substrate oxidation test experiment was set up similarly, but 20 µL of etomoxir (Eto, 4 µM final concentration) was added to port A of the cartridge. The other ports were loaded as described for the Mito Stress kit, and the analysis was run likewise.

### 2.7. AdipoRed Staining

After 1 and 13 days of incubation and treatment, DMEM was removed, and each well was washed with DPBS (+Ca/Mg). Subsequently, paraformaldehyde was added for cell fixation, and the plates were stored at 4 °C. Next, intracellular lipids were stained with AdipoRed 2.5% solution, diluted in DPBS at a 1:40 ratio for 30 min. Finally, the IncuCyte was used to create phase-contrast and red fluorescence images and to calculate the red fluorescence intensity per surface covered with cells.

### 2.8. Periodic Acid–Schiff (PAS) Staining

Similarly, cells fixed in paraformaldehyde were washed with distilled water and treated with periodic acid (0.5%) for 5 min. Next, the cells were washed three times with distilled water, and Shiff’s reagent was added for 15 min. After incubation, the remaining Shiff’s reagent was washed away with water, and the cells were imaged using a Zeiss Primovert phase-contrast microscope with an Axiocam color camera.

### 2.9. Statistics

Statistical analysis of the resazurin and SRB cytotoxicity assays, as well as the AdipoRed assay and maximal respiration after Eto injection, was performed using SPSS Statistics 29 software. A two-tailed Student’s *t*-test was applied to compare treated samples wit h untreated controls. Comparisons between the untreated control and the different iminosugar treatments for each mitochondrial parameter (basal respiration, maximal respiration, ATP production, proton leakage, non-mitochondrial respiration, and spare respiratory capacity) were performed using one-way analysis of variance (ANOVA), followed by Tukey’s HSD post hoc test to identify statistically significant differences (*p* < 0.05). The extracellular acidification rate parameter was also statistically analyzed using one-way ANOVA followed by Tukey’s HSD post hoc test. These analyses were carried out using GraphPad Prism version 8. For iminosugar-treated samples, the number of replicates ranged from n = 4 to n = 5; for untreated controls, from n = 7 to n = 31.

## 3. Results

### 3.1. Iminosugars Affect Cell Viability of Intestinal Cell Lines

In the first step, cell viability after exposure to iminosugars was investigated in the Caco-2 and HCT-116 cell lines, representing the early and late stages of colon cancer, according to the ATCC product files. Table 1 shows the viability of cell monolayers exposed to iminosugars for the short term (24 h) in either a glucose-rich or glucose-depleted medium.

**Figure 2:**
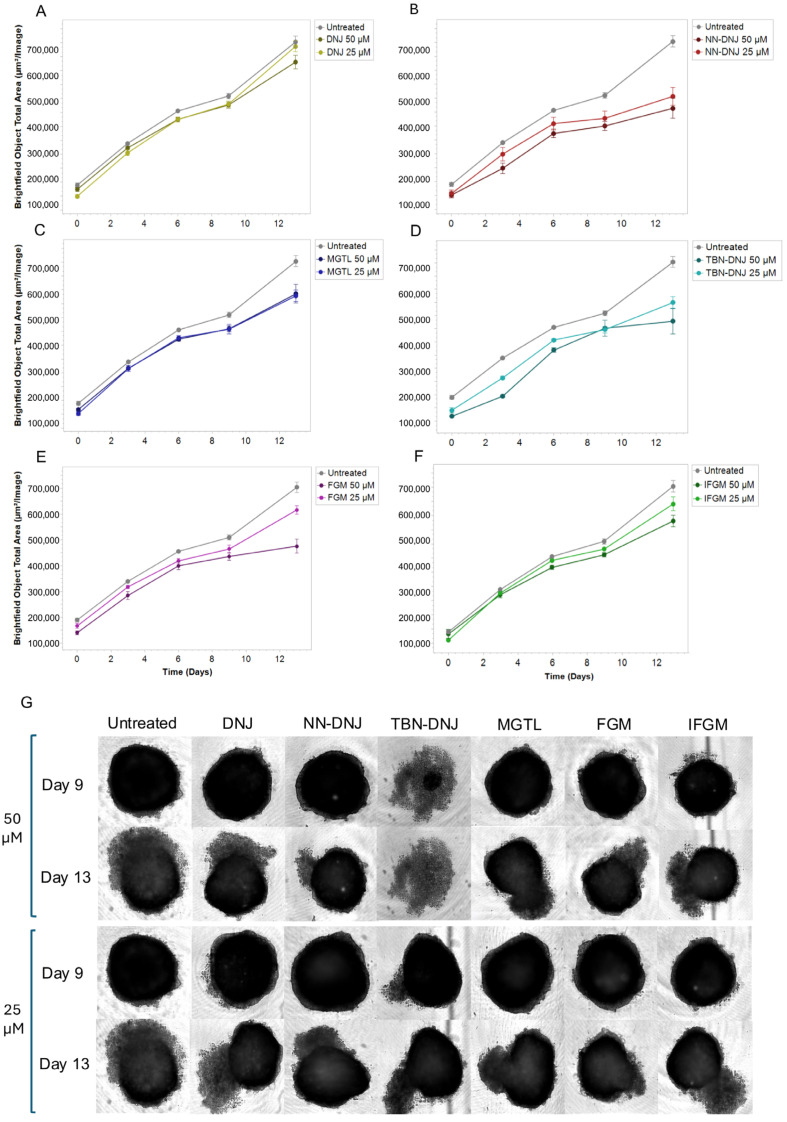
Effects of treating HCT-116 cells with iminosugars on spheroid growth over time. (**A**–**F**) The graphs represent growth curves of cells when treated with the iminosugars (**A**) DNJ; (**B**) NN-DNJ; (**C**) TBN-DNJ; (**D**) MGTL; (**E**) FGM; and (**F**) IFGM; or untreated, over a time frame of 0 to 13 days, obtained using the IncuCyte live-cell imaging system, which captures phase-contrast images over time. The images depict spheroids on days 9 and 13 after treatment with either 50 μM or 25 μM iminosugar (**G**). The images illustrate the evolution over time of the morphology and cellular density of the spheroids.

Generally, treatment of cells with iminosugars resulted in a lower mitochondrial activity (resazurin), protein content (SRB), and confluency compared to untreated cells. The effects varied depending on the type of iminosugar, cell type, and growth conditions (presence or absence of glucose). Particularly in the Caco-2 cell line, treatment with DNJ derivatives in the absence of glucose generally showed lower values compared to treatments with glucose, indicating a possible metabolic dependence on glucose for maintaining cell viability and proliferation. Notably, in a comparison of the natural iminosugars, FGM appeared less impactful than DNJ. Among the synthetic derivatives, TBN-DNJ and NN-DNJ showed a more negative impact on cell confluence, suggesting potential cytotoxic or inhibitory effects on proliferation. In the next step, the effect of iminosugar treatment on a 3D spheroid model, which better represents tumor architecture, was investigated (Figure 2). HCT-116 cells were used because Caco-2 cells are unable to form such 3D structures, and an exposure time of 13 days was applied to allow sufficient tumor growth and simulate longer-term treatment. Growth curves monitored via the IncuCyte live cell imager showed that NN-DNJ, MGTL, TBN-DNJ, and FGM were more effective in decreasing spheroid diameter compared to 1-DNJ and IFGM.

### 3.2. Iminosugars Affect Energy Metabolism in HCT-116-Derived Spheroids

**Figure 3:**
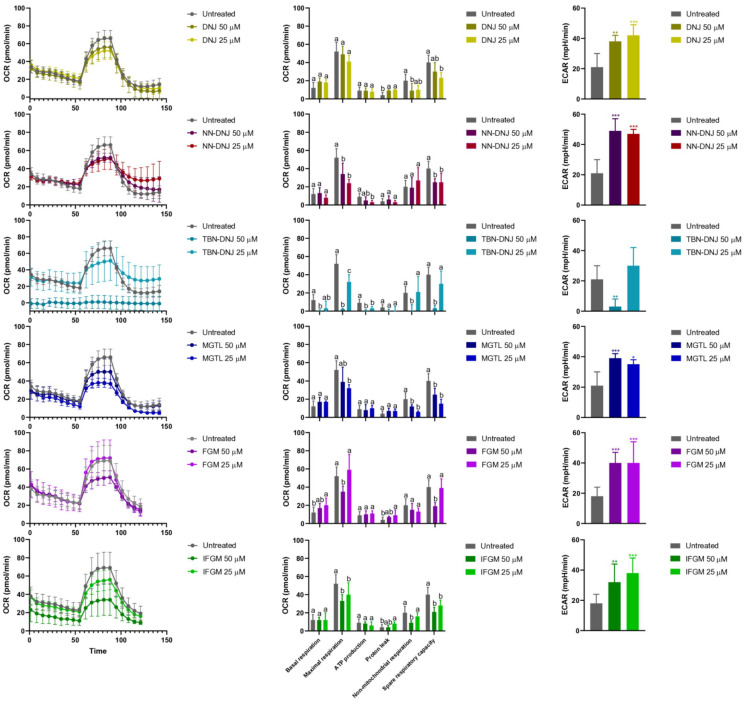
The effect on energy production, as determined by the XFe96 Efflux Analyzer (Agilent Technologies, Diegem, Belgium), is given for the treatment of cells with 50 μM or 25 μM iminosugar. Left panels show OCR as a function of time and upon different stressor injections. The middle panels show various mitochondrial parameters. Basal respiration refers to mitochondrial activity at rest. Maximal respiration after the injection of FCCP, an uncoupling agent that forces the electron transport chain to work at its full capacity, indicates mitochondrial reserve capacity. Mitochondrial ATP production is the difference between basal respiration and the OCR after OM inhibition of the ATP synthase. Proton leakage is the residual OCR after OM addition, and it reflects oxygen consumption not linked to ATP production but rather due to proton leakage across the mitochondrial membrane. Non-mitochondrial respiration is the OCR after the complete inhibition of the electron transport chain by R/AM and reflects oxygen consumption by non-mitochondrial sources (e.g., oxidases or peroxisomes). Spare respiratory capacity is the difference between maximal and basal respiration, representing the highest possible reserve capacity upon elevated energy demand. A low value may indicate that cells are already operating near their respiratory limit, making them more vulnerable to stress. On the right, ECAR in spheroids after 13 days of treatment is depicted. Data are presented as mean ± standard deviation. Mean values marked with different letters (i.e., a, b, c) for each mitochondrial parameter indicate significant differences (*p* < 0.05) among treatments, as determined by one-way ANOVA followed by Tukey’s HSD post hoc test. Statistical difference is marked by ***: *p* < 0.001, **: *p* < 0.01, *: *p* < 0.05 compared with control by ANOVA followed by Tukey’s HSD post hoc test.

To understand the effect of iminosugars on tumor growth, the energy metabolism was investigated closely as iminosugars are specific inhibitors of glucosidase activity, potentially impacting glucose and other energetic substrate mobilization within cells. To this end, spheroids were transferred to a 96-well spheroid plate, and shifts in energy metabolism were monitored using the XFe96 Efflux Analyzer. Figure 3 shows that basal respiration, reflected by the oxygen consumption rate (OCR) before the addition of mitochondrial stressors, was maintained upon treatment with most iminosugars, except TBN-DNJ at 50 µM. Despite maintained basal respiration, a higher glycolytic rate, indicated by a higher extracellular acidification rate (ECAR), was observed, suggesting a shift to a less efficient energy metabolism pathway to meet energy demands. Additionally, for all tested conditions, maximal respiratory capacity, visible after FCCP injection, was lower than that in the untreated condition, indicating an impact on mitochondrial fitness. Interestingly, the maximal respiration of cells treated with 25 µM FGM was higher than that of all other treatments.

As shown in Figure 3, bioenergetic analysis of spheroids treated with DNJ, FGM, and their synthetic derivatives for 13 days showed a decrease in the oxygen consumption rate, most pronounced at 50 μM iminosugar. Mitochondrial parameters revealed that basal respiration was generally similar between treated and untreated spheroids. However, a decrease in both maximal respiration and spare respiratory capacity, defined as the mitochondria’s ability to meet increased energy demands, was observed. Significantly decreased non-mitochondrial respiration for various treatments (except FGM and NN-DNJ), particularly at 50 µM, may point to a decreased overall viability. In general, no substantial differences were observed for mitochondrial ATP production or proton leakage. Interestingly, the extracellular acidification rate was significantly increased for all iminosugar treatments, indicating a shift to a less efficient compensatory mechanism to meet energy demands. As an exception, TBN-DNJ significantly decreased the extracellular acidification rate at 50 µM, likely due to a higher cytotoxicity compared to other compounds. Overall, strong shifts in energy metabolism occurred, with many natural and synthetic iminosugars inducing similar effects in terms of maximal respiration and glycolysis, leading to increased lactate production, a key feature of aerobic glycolysis often observed in tumor cells in the presence of oxygen.

### 3.3. Iminosugars Induce Shifts in Substrate Use for Energy Metabolism

**Figure 4:**
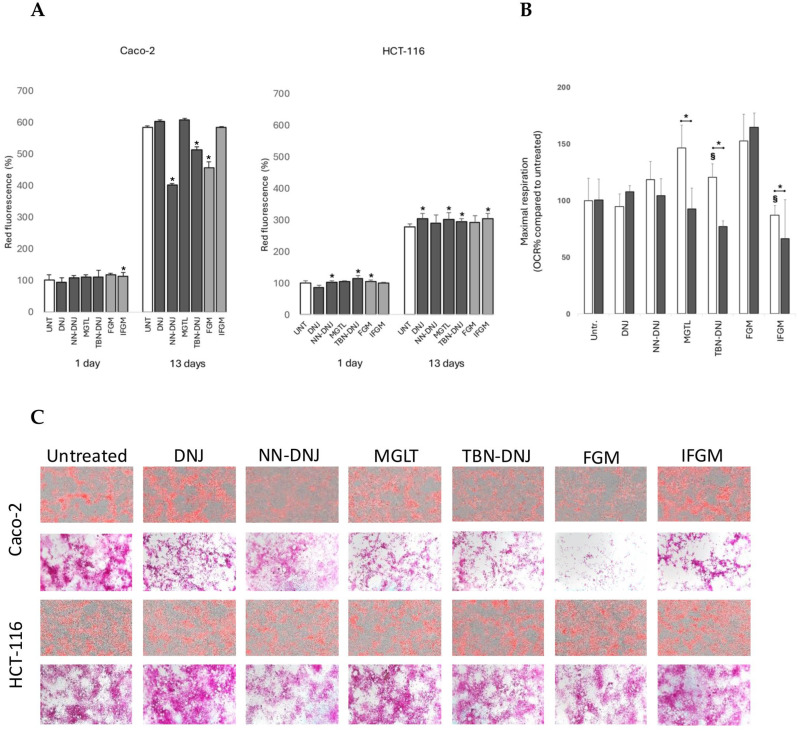
Impact of iminosugars on nutrient use in colon cancer cells. (**A**) Intracellular lipid content (AU) upon AdipoRed staining of Caco-2 cells. *: indicates significant difference compared to the untreated control (*p* < 0.05). (**B**) Maximal respiration of HCT-116 spheroids treated for 13 days with iminosugars after injection of the fatty acid transport inhibitor Eto. *: significantly different after Eto injection of the same iminosugar treatment, §: from the untreated condition (*p* < 0.05). (**C**) Microscopic pictures of Caco-2 and HCT-116 cells treated with iminosugars for 13 days after AdipoRed staining for lipids (**upper**) and PAS staining for glycogen (**lower**).

To further investigate whether these shifts in metabolism could result from differences in nutrient use for respiration, the major energy stores of the cells, glycogen and lipids, were stained microscopically. Additionally, using Eto as a carnitine palmitoyltransferase-1 inhibitor to inhibit fatty acid transport into the mitochondria, we aimed to further distinguish between the modes of action of natural and synthetic iminosugars. Overall, quantification of the AdipoRed stain as a proxy for intracellular lipid content showed increased values over time (Figure 4A). While no significant differences were observed in the early stages, marked variations emerged by day 13. In Caco-2 cells, a significant decrease in intracellular lipid content was observed upon long-term treatment with NN-DNJ, TBN-DNJ, and FGM compared to the untreated control. Interestingly, such a decrease was not visible in long-term-treated HCT-116 cells. Figure 4B shows that maximal mitochondrial respiration in HCT-116 spheroids (13 days) was affected by iminosugar treatment and Eto injection. Spheroids treated with MGTL, TBN-DNJ, and IFGM showed increased dependence on lipids for mitochondrial respiration. Microscopic pictures of PAS staining for glycogen in Caco-2 cells treated for 13 days (Figure 4C) showed a strong reduction in glycogen content in cells treated with iminosugars, particularly FGM. This suggests that certain iminosugars influence lipid metabolism and cellular glycogen storage, likely due to their inhibitory effect on *α*-glycosidases. Additionally, glycogen distribution in different zones was diverse in untreated conditions, whereas iminosugar treatment resulted in either a more diffuse and equally distributed phenotype (NN-DNJ) or more pronounced site-specific localization (IFGM). These effects were less pronounced in HCT-116-treated monolayers (Figure 4C). Similarly, intracellular lipid concentration followed the same distribution pattern in cell monolayers, indicating a role of iminosugars in cell differentiation and epithelial organization.

## 4. Discussion

This study presents, to our knowledge, the first attempt to investigate the effects of natural iminosugars and synthetic derivatives on two intestinal cell lines by integrating innovative techniques such as three-dimensional culture techniques and bioenergetic phenotyping. Although multiple studies have demonstrated the impact of drug-like iminosugars, such as MGLT and FGM, on human metabolism, almost no studies compare the effectiveness and compound-specific effects in a single screening. Our results demonstrate that both natural and synthetic iminosugars (i) decrease cell growth depending on the iminosugar, the presence of glucose in the medium, and the cell type and architecture, (ii) decrease maximal respiration and increase glycolysis, indicating a less efficient energy production machinery, (iii) alter substrate preferences to sustain mitochondrial respiration, and (iv) substantially alter lipid and glycogen deposits in intestinal monolayers depending on the cell type and iminosugar.

### 4.1. Iminosugars Decrease Cell Growth Depending on Iminosugar Structure, Presence of Glucose in the Medium, and Cell Type and Architecture

Our findings indicate that iminosugars significantly impact cellular bioenergetics, particularly affecting the cells’ ability to cope with metabolic stress and increased energy demands. The strength of this effect varied depending on the type and concentration of the iminosugar, with the most pronounced impairments observed for synthetic derivatives at 50 µM. Overall, cell growth was inhibited by iminosugars, and this effect was enhanced under glucose-deprived conditions compared to glucose-rich conditions. This aspect is likely attributable to one of the main characteristics of iminosugars, namely their ability to inhibit key enzymes regulating the availability of energy substrates. As iminosugars act as glycosidase inhibitors, the mobilization of glycogen as a backup nutrient in case of glucose depletion is hampered. This feature may be beneficial for targeting cancer cells located at the inner core of the tumor, where nutrient and oxygen depletion is more prevalent than in the outer layer. Hampering glycolysis in cancer cells can also contribute to combating drug resistance by reducing the available energy for multidrug efflux pumps [15]. Additionally, the inhibition of *α*-glucosidase and mannosidase can lead to a reduction in glycolysis and protein synthesis, as well as increased endoplasmic reticulum stress [16], which may lead to cytotoxic effects. Furthermore, as glycosidases are important enzymes for tumor cell surface glycoconjugate synthesis, their inhibition may alter the 3D architecture of the tumor spheroid, making the cells more loosely connected to each other, as observed with TBN-DNJ. This hypothesis should be confirmed by determining specific cell surface glycoconjugates using more complex analytical techniques such as chromatography (lectin) coupled with mass spectrometry.

In the presence of glucose, natural compounds such as FGM showed a positive effect on mitochondrial activity, as reflected by higher resazurin conversion compared to the control in both Caco-2 and HCT-116 monolayers in the short term, as well as by higher maximal respiration in HCT-116 spheroids in the long term. This effect may suggest a protective and stimulatory capacity for cell metabolism under conditions of sufficient energy availability. However, more pronounced and inhibitory effects on cell viability were observed with synthetic derivatives compared to natural iminosugars, especially at higher concentrations. For instance, the higher effects with TBN-DNJ and NN-DNJ suggest that chemical modifications, i.e., the hydrophobic side chains introduced in synthetic derivatives, may improve the uptake of iminosugars by passive diffusion through cell membranes [17,18]. In addition, the aromatic rings in the structure of TBN-DNJ introduce extra features, possibly improving the iminosugar’s affinity for enzymatic targets, enhancing uptake through cellular receptors, or increasing the metabolic stability of the compound.

### 4.2. Iminosugars Decrease Maximal Respiratory Capacity and Increase Glycolysis

This research is, to our knowledge, the first report investigating the effect of iminosugars on cellular bioenergetics. Our initial observation was that treatment with different iminosugars led to decreased basal respiration compared to untreated cells. This decrease was particularly visible at higher concentrations (50 µM), suggesting that these compounds may interfere with mitochondrial respiratory capacity. Interestingly, natural DNJ and FGM had a less pronounced effect compared to their synthetic derivatives, indicating that structural modifications in the synthetic derivatives may be more effective in targeting energy metabolism or may have better stability and cellular uptake. Similarly, except for DNJ, all iminosugars were able to reduce the spare mitochondrial capacity after FCCP stress. This suggests that either fewer mitochondria were present or that shifts in mitochondrial enzymes and/or structure were induced by the iminosugars. Indeed, the literature reports that iminosugars can inhibit glucocerebrosidase, which may affect lipid metabolism within the mitochondria. This effect was demonstrated for DNJ analogs such as N-butyl-1-deoxynojirimycin (N-Butyl-DNJ), which is investigated for treating Gaucher’s disease, and N-dodecyl-1-deoxynojirimycin (N-dodecyl-DNJ) [19,20].

Our results also demonstrated that this decrease in mitochondrial capacity was paralleled by an increase in glycolysis and lactate production for all tested iminosugars, suggesting that cells may become more dependent on alternative energy pathways to sustain their survival and to compensate for their energy deficit apart from oxidative phosphorylation. Whether this is a beneficial effect is still debatable. On one hand, stimulation of this inefficient pathway for ATP production may limit the outgrowth of cancer cells and hence open opportunities for iminosugars to be used in cancer treatment. On the other hand, the shift towards glycolysis, also called the Warburg effect, is a typical hallmark for cancer progression. This metabolic state provides greater plasticity, allowing cancer cells to adapt to different microenvironments [21]. More specifically, the Warburg effect offers tumor cells higher flexibility in nutrient utilization, enabling them to metabolize various energy substrates to meet the increasing bioenergetic and biosynthetic demands of tumor development across diverse microenvironments. Additionally, intermediate metabolites of glycolysis, such as lactate and pyruvate, can be recycled for the synthesis of essential cellular biomass, thus promoting cell proliferation. Furthermore, the shift towards glycolysis may limit the overproduction of reactive oxygen species (ROS), which may trigger adaptive stress responses that support survival, tumor progression, and mutagenic events promoting tumorigenesis and metastasis, while avoiding the cytotoxic effects associated with excessive ROS levels. Therefore, the hybrid metabolic phenotype has been specifically associated with metastatic potential, supporting the ability of cancer cells to adapt to and colonize distant environments [22].

As we are the first to report this shift towards glycolysis, it is not yet clear whether this effect is beneficial for the cell, although it is now known that lactate is used continuously as fuel by various cells under conditions of complete aerobiosis. Under low-glucose conditions, cancer cells take up and oxidize lactate [23]. Enhanced glycolysis could be the result of endoplasmic stress, as various glycosidases are crucial in protein glycosylation and quality control, or it can be a sign pointing towards autophagy [24,25]. It is likely that the higher glycolytic rate is not really meant to compensate for increased energy demands, but may simply be a compensatory side effect of the cells maintaining the production rate of essential glycoconjugates due to the inhibition of glycosidase activity [26,27]. To support this hypothesis, it would be relevant to examine potential changes in nutrient transporters and mitochondrial and related enzymes, as well as in the glycoconjugate profile of the cells.

Overall, we may conclude that the actual effect of iminosugars on the induction of a glycolytic shift has not yet been clearly demonstrated. To date, no studies in the literature have directly shown an association between these compounds and the modulation of glycolytic metabolism. However, since iminosugars inhibit glycosidases, thereby altering the glycosylation profile of numerous proteins, including those involved in the regulation of cellular metabolism, it is plausible to hypothesize an indirect effect on metabolic pathways, which deserves further experimental investigation.

### 4.3. Iminosugars Alter Lipid and Glycogen Deposits in Intestinal Monolayers and Affect Tissue Organization

Our results clearly show that long-term iminosugar treatment impacts nutrient deposits in cells, depending on the iminosugar structure and cell line. The most pronounced differences were observed in differentiating Caco-2 cells. Microscopic staining of intracellular lipids revealed that cells treated with NN-DNJ, TBN-DNJ, and FGM had significantly lower intracellular lipid content compared to control cells. This suggests that, in response to iminosugars, the cells may exhibit decreased lipogenesis, increased fatty acid consumption as a secondary energy source, and/or effects on other pathways such as the inhibition of glucocerebrosidases. To test whether intracellular fatty acids were used to compensate for energy deficits, etomoxir was added as an inhibitor of fatty acid transport into mitochondria. A significant decrease in maximal respiration was confirmed for the slightly cytotoxic TBN-DNJ but not for NN-DNJ and FGM. Additionally, MGLT and IFGM showed higher dependency on fatty acid oxidation for energy metabolism. We therefore concluded that increased fatty acid oxidation is likely not the primary pathway explaining the lower lipid deposits in cells caused by NN-DNJ and FGM.

In the literature, DNJ consumption has been associated with decreased triglyceride content in the liver and increased fatty acid *β*-oxidation [28]. However, in our intestinal cell models, only synthetic DNJ derivatives showed a similar effect. Whether this is due to the stability of DNJ or differences in DNJ uptake or function in other tissue types remains unclear and warrants further study. DNJ has also been reported to inhibit acetyl-*Co*A carboxylase and fatty acid synthase, which may directly decrease lipogenesis. Furthermore, in fat tissue, adiponectin and AMPK were upregulated. Together with a decrease in lipogenesis, this may have beneficial effects on atherosclerosis and fatty liver disease. Whether such effects also occur in intestinal cells needs to be established.

In the literature, DNJ and FGM analogs, such as N-butyl-DNJ and N-dodecyl-DNJ, are reported to inhibit glucocerebrosidase, thereby affecting (sphingo)lipid metabolism in Gaucher disease. The accumulation of substrates such as glucosylceramide, which impairs mitochondrial function and autophagy and induces the hyperactivation of the mTORC1 pathway in neuronal models, suggests a broader impact on cellular homeostasis beyond lipid storage [29]. It can therefore be suggested that NN-DNJ, TBN-DNJ, and FGM have a similar mode of action in differentiating cells. Sphingolipids are constituents of the cell membrane, modulators of cellular trafficking, and precursors for mediators and messenger molecules. They play an important role in gut homeostasis, intestinal barrier integrity, immune responses, and composition of the gut microbiome [30]. Additionally, sphingolipids are important mediators of cancer cell growth, migration, transformation, and drug resistance [31,32]. In particular, ceramide, the end product of glucocerbrosidase, has a beneficial effect against cancer by inhibiting many of these processes. Therefore, sphingolipid dynamics should be studied before considering the use of iminosugars to treat intestinal diseases and cancer.

Furthermore, staining of intracellular glycogen indicated that iminosugars influence not only lipid but also glycogen metabolism. Glycogen is an intracellular polymer consisting of *α*-(1,4) and *α*-(1,6) bound glucose units. Its synthesis requires glucose transport inside the cell and multiple phosphorylation and isomerization reactions to generate UDP-glucose as a donor. On the other hand, glycogen is mobilized by an interplay of glycogen phosphorylases, glycogen detangling enzyme and lysosomal degradation by *α*-glycosidases, and the glucose-6-phosphatase system [33]. In Caco-2 cells treated for 13 days, a visible reduction in glycogen accumulation was observed in cells treated with all tested iminosugars, especially FGM. The literature reports that FGM can reduce postprandial blood glucose levels upon intake of sucrose and starch by inhibiting disaccharidase activity [34]. Likewise, the antihyperglycemic action of MGLT is caused by a reversible inhibition of membrane-bound intestinal *α*-glucoside hydrolase enzymes [35]. Membrane-bound intestinal α-glucosidases hydrolyze oligosaccharides and disaccharides to glucose and other monosaccharides in the brush border of the small intestine. Yet, in our cell culture setup, such brush border disaccharidase activity is not needed as monomeric glucose is the only external sugar source. Furthermore, if iminosugars such as FGM and MGTL would inhibit intracellular *α*-glycosidases responsible for glycogen mobilization, we would expect a higher instead of a lower glycogen content. Such an increase in glycogen content, especially in liver and muscle tissues, was reported upon DNJ administration to a diabetic tilapia model [36]. Although in normal tilapia, no changes in the activity of enzymes involved in glycogen synthesis were observed, DNJ caused a significant increase in hexokinase, glucose-6-phosphate dehydrogenase, glucose-6-phosphatase, and fructose 1,6-biphosphase. The glycogen content and these enzymatic activities were not studied in intestinal tissue, so it remains unknown how iminosugars affect glucose dynamics in the intestinal cells.

Microscopic images not only revealed differences in lipid and glycogen content in Caco-2 cells but also showed three distinct phenotypes in monolayer organization: (i) a high distribution of nutrients in both flat and elongated cells (untreated), (ii) a low distribution in flat cells while a high content was maintained in elongated cells (DNJ, MGLT, TBN-DNJ, IFGM), and (iii) a general ‘flattening’ of the monolayer with relatively low and more equal distribution (NN-DNJ and FGM). These altered phenotypes of epithelial monolayers may indicate important changes in differentiation and tissue architecture. Indeed, DNJ has been shown to inhibit the expression of MMP2 and MMP9, upregulate TIMP-2, and alter cell surface glycan-binding motifs, which are crucial in extracellular matrix remodeling, differentiation, and migration of tumor-derived cells [37].

One important observation was that the more tumorigenic and metastatic HCT-116 cell line was less susceptible to changes in lipid and glycogen accumulation, and the cell monolayer was less differentiated compared to the long-term-treated Caco-2 cell line. This may be due to the fact that cancer cells inherently have higher lipid and glycogen stores to meet the higher demand for substrates for energy production and biosynthesis of rapidly dividing cells, to overcome changing tissue environments, cope with oxidative stress, and facilitate cell–cell signaling to promote cell proliferation [38].

## 5. Conclusions

This study provides valuable insights into the effects of various iminosugars on cellular energy metabolism, nutrient distribution, and tissue architecture, representing a significant advancement in this field. A thorough review of the available literature did not identify previous studies specifically addressing this issue, highlighting the originality and relevance of the present work. By filling this gap, our results offer a novel contribution to the field, opening new perspectives for future research aimed at understanding how iminosugars, both natural and synthetically derived, influence cellular bioenergetics.

In particular, this study demonstrates that iminosugars alter cell growth in colon cancer Caco-2 and HCT-116 cells in a sugar- and environment-specific way, reducing their basal respiration and spare mitochondrial capacity while increasing glycolysis and lactate production. Moreover, long-term treatment induces changes in the substrate preferences for energy metabolism and alters lipid and glycogen deposits in intestinal monolayers. These changes in nutrient deposits are accompanied by alterations in tissue organization.

Despite these promising results, our study has some limitations. In vitro models, although representative, cannot fully replicate the complexity of the biological environment in vivo. Therefore, future studies should focus on clinical trials to further investigate the systemic effects of iminosugars in humans. In conclusion, this study demonstrates the potential of iminosugars in modulating cellular metabolism, opening new perspectives for cancer treatment. Future research should focus on dose optimization, safety assessment, and the integration of these compounds into innovative therapeutic approaches.

## Figures and Tables

**Table 1:** The effect of natural iminosugars and synthetic derivatives on the growth and metabolism of Caco-2 and HCT-116 cell lines after 24 h of incubation at a concentration of 50 µM in DMEM with and without glucose. Cellular parameters were assessed using the resazurin metabolic activity assay (Res.), the protein content assay (SRB), and cell confluency (IncuCyte). Results are expressed as mean ± standard deviation (SD), with statistical significance indicated by an asterisk (*) relative to untreated controls (*p* < 0.05). Statistical significance was determined using a two-tailed *t*-test between treated samples and untreated controls.

Iminosugar	Glucose	Caco-2			HCT-116		
	+/−	Mitochondrial Activity	Protein Content	Confluence	Mitochondrial Activity	Protein Content	Confluence
DNJ	+	103.8 ± 15.2	59.1 ± 14.2 *	89.9 ± 6.6	114.7 ± 17.1	86.8 ± 14.5	44.4 ± 6.5 *
	−	87.9 ± 5.9 *	52.3 ± 7.2 *	58.7 ± 5.8 *	74.8 ± 17.3 *	79.4 ± 17.1 *	88.8 ± 3.9
NN-DNJ	+	91 ± 14.9	65.3 ± 11.8 *	65.2 ± 11 *	89.3 ±21.6	72.9 ± 15.9 *	58.9 ± 4.5 *
	−	86.4 ± 11.5 *	54.6 ± 6.1 *	50.8 ± 12.3 *	83.9 ± 15.5	87.3 ± 7.4	78.2 ± 10.7
TBN-DNJ	+	82.9 ± 7.9	68.28 ± 16.1 *	56.5 ± 12.6 *	66.5 ± 21 *	61.56 ± 8.8 *	54 ± 9.5 *
	−	22.1 ± 5.4 *	40.65 ± 15.2 *	36.2 ± 5.1 *	49.9 ± 3.7 *	65.21 ± 3.7 *	56 ± 9.8 *
MGTL	+	87.4 ± 7.5 *	109.15 ± 21.3	79.6 ± 17.6	77.9 ± 14.2	107.3 ± 18	93.6 ± 18.7
	−	93 ± 4.4 *	67.9 ± 9.6 *	72.4 ± 14.7 *	74.8 ± 10.1 *	74.71 ± 13.2 *	88.6 ± 13.4
FGM	+	126.2 ± 24.3 *	79.7 ± 21.5	100.2 ± 9.7	104.3 ± 15.1	99.4 ± 6.7	91.1 ± 9.4
	−	126.8 ± 27	72.6 ± 17.3 *	82.1 ± 14.2 *	92.8 ± 10.7	97.3 ± 21.4	121.6 ± 15.2
IFGM	+	94.4 ± 11.6	89.37 ± 16.5	80.3 ± 9.9 *	85.3 ± 13.9	93.5 ± 9.1	92.4 ± 10.4
	−	110.6 ± 13.4	71.26 ± 19	69.1 ± 10.4 *	76.2 ± 9.9 *	80 ± 21.5	70.7 ± 11.5 *

## Data Availability

The original data presented in the study are openly available in Zenodo at https://doi.org/10.5281/zenodo.15310495 (accessed on 30 April 2025).

## Abbreviations

DNJ	1-deoxynojirimycin
FGM	D-fagomine
IFGM	Isofagomine
MGTL	Miglitol
NN-DNJ	N-(n-Nonyl)deoxynojirimycin
TBN-DNJ	2,3,4,6-Tetra-O-benzyl-(+)-1-deoxynojirimycin
ASSPC	Active-site-specific pharmacological chaperone
DMEM	Dulbecco’s modified Eagle’s medium
DPBS	Dulbecco’s phosphate-buffered saline
FBS	Fetal Bovine Serum
SRB	Sulforhodamine B
ETC	electron transport chain
OM	Oligomycin
FCCP	Carbonyl cyanide-p-trifluoromethoxyphenylhydrazone
R/AM	Rotenone/Antimycin
Eto	Etomoxir
PAS	Periodic acid–Schiff
ANOVA	One-way analysis of variance
Res.	Resazurin metabolic activity assay
SD	Standard deviation
OCR	Oxygen consumption rate
ECAR	Extracellular acidification rate
N-Butyl-DNJ	N-butyl-1-deoxynojirimycin
N-dodecyl-DNJ	N-dodecyl-1-deoxynojirimycin
ROS	Reactive oxygen species
